# Supplementary material for: The Determinants of the 13-Year Risk of Incident Atrial Fibrillation in a Russian Population Cohort of Middle and Elderly Age
Source: J Pers Med. 2022 Jan 17;12(1):122. doi: 10.3390/jpm12010122 (PMC8779704; doi:10.3390/jpm12010122)
Supplement: Supplementary file 1 [file jpm-12-00122-s001.zip › jpm-1527415-supplementary.pdf]

Journal of *Personalized Medicine*

MDPI

Supplementary Materials:

Supplementary Table S1. Associations between risk factors and 13-risk of incident AF. Cox-regression analysis, age- and multivariable-adjusted models (The HAPIEE study, Russian population cohort free from baseline CVD, n=3871).

Risk Factors	Model 1<br>HR (95% CI)	Model 2<br>HR (95% CI)	Model 3<br>HR (95% CI)
Age, per 1 year	**1,10 (1.07-1.13)**	**1.09 (1.06-1.12)**	**1.10 (1.07-1.14)**
Sex: male vs. female	**1.95 (1.36-2.79)**	**2.02 (1.17-3.47)**	**2.20 (1.26-3.87)**
Heart rate, per 1 b / min	1.00 (0.98-1.02)	0.99 (0.98-1.01)	
BMI, per 1 kg/m^2	**1.10 (1.07-1.14)**	**1.10 (1.06-1.15)**	**1.11 (1.07-1.15)**
SBP, per 1 mmHg	**1.02 (1.01-1.02)**	**1.02 (1.01-1.02)**	
DBP, per 1 mmHg	**1.03 (1.01-1.04)**		
TC, per 1 mmol/L	**0.81 (0.69-0.95)**	**0.79 (0.66-0.95)**	**0.79 (0.67-0.94)**
LDLC, per 1 mmol/L	**0.79 (0.66-0.94)**		
HDLC, per 1 mmol/L	0.78 (0.45-1.35)		
TG, per 1 mmol/L	0.97 (0.76-1.23)		
Glucose, per 1 mmol/L	0.91 (0.76-1.09)		
GGTP, per 1 U/L	1.00 (1.00-1.01)		
HT, yes vs. no	**1.75 (1.15-2.65)**		1.50 (0.97-2.33)
DM, yes vs. no	0.94 (0.49-1.79)		0.83 (0.43-1.6)
Alcohol, per 20 g per drinking occasion	1.22 (1.02-1.22)	1.00 (1.00-1.01)	1.0 (1.00-1.01)
Smoking status:			
- former smoker vs. never	1.20 (0.68-2.11)	1.21 (0.68-2.14)	1.18 (0.66-2.09)
-current smoker vs. never	1.18 (0.71-1.98)	1.39 (0.81-2.40)	1.14 (0.80-2.35)
Level of education:			
- other vs. higher	1.18 (0.80-1.73)		1.14 (0.76-1.70)
Marital status:			
- single vs. married	1.18 (0.77-1.81)		1.15 (0.73-1.79)

HR - hazard ratio, CI - confidence interval. Model 1: age- and sex- adjusted; Model 2: adjusted for age, sex, BMI, SBP, TC, TG, smoking, alcohol consumptions. Model 3: for age, sex, BMI, TC, HT, DM, smoking, alcohol consumptions, education and marital status

Supplementary Table S2. Associations between risk factors and 13-risk of incident AF. Cox-regression analysis, age- and multivariable-adjusted models (The HAPIEE study, Russian population cohort, n=5759).

Risk Factors	Model 1<br>HR (95% CI)	Model 2<br>HR (95% CI)	Model 3<br>HR (95% CI)
Age, per 1 year	**1.09 (1.06-1.12)**	**1.09 (1.06-1.12)**	**1.10 (1.07-1.14)**
Sex: male vs. female	**1.96 (1.37-2.79)**	**1.94 (1.18-3.18)**	**2.20 (1.25-3.85)**
CVD at baseline	**2.48 (1.74-3.38)**	**2.22 (1.57-3.34)**	**2.25 (1.59-3.17)**
Heart rate, per 1 b / min	1.00 (0.98-1.01)	0.99 (0.97-1.01)	
BMI, per 1 kg/m^2	**1.10 (1.06-1.14)**	**1.09 (1.05-1.13)**	**1.10 (1.07-1.14)**
SBP, per 1 mmHg	**1.02 (1.01-1.02)**	**1.02 (1.01-1.02)**	
DBP, per 1 mmHg	**1.03 (1.02-1.04)**		
TC, per 1 mmol/L	**0.80 (0.68-0.94)**	**0.76 (0.64-0.90)**	**0.77 (0.65-0.91)**
LDLC, per 1 mmol/L	**0.78 (0.65-0.93)**		
HDLC, per 1 mmol/L	0.87 (0.51-1.49)		
TG, per 1 mmol/L	0.96 (0.76-1.23)		
Glucose, per 1 mmol/L	0.90 (0.73-1.08)		
GGTP, per 1 U/L	1.00 (1.00-1.01)		
HT, yes vs. no	**1.73 (1.14-2.62)**		1.47 (0.95-2.27)
DM, yes vs. no	1.12 (0.60-2.35)		1.34 (0.67-2.27)
Alcohol, per 20 g per drinking occasion	1.08 (0.98-1.20)	1.06 (0.96-1.17)	1.04 (0.94-1.15)
Smoking status:			
- former smoker vs. never	1.20 (0.68-2.11)	1.21 (0.68-2.12)	1.17 (0.66-2.10)
-current smoker vs. never	1.15 (0.69-1.93)	1.35 (0.79-2.31)	1.13 (0.77-2.30)
Level of education:			
- other vs. higher	1.17 (0.80-1.72)		1.13 (0.76-1.70)
Marital status:			
- single vs. married	1.17 (0.77-1.83)		1.16 (0.74-1.81)

HR - hazard ratio, CI - confidence interval. Model 1: age- and sex- adjusted; Model 2: adjusted for age, sex, BMI, SBP, TC, TG, smoking, alcohol consumptions, baseline CVD. Model 3: for age, sex, BMI, TC, HT, DM, smoking, alcohol consumptions, education and marital status, baseline CVD
